# The multivesicular body is the major internal site of prion conversion

**DOI:** 10.1242/jcs.165472

**Published:** 2015-04-01

**Authors:** Yang-In Yim, Bum-Chan Park, Rajgopal Yadavalli, Xiaohong Zhao, Evan Eisenberg, Lois E. Greene

**Affiliations:** Laboratory of Cell Biology, NHLBI, NIH, Bethesda, MD 20892, USA

**Keywords:** Conversion, Multivesicular body, Prion, Scrapie

## Abstract

The conversion of the properly folded prion protein, PrPc, to its misfolded amyloid form, PrPsc, occurs as the two proteins traffic along the endocytic pathway and PrPc is exposed to PrPsc. To determine the specific site of prion conversion, we knocked down various proteins in the endocytic pathway including Rab7a, Tsg101 and Hrs (also known as HGS). PrPsc was markedly reduced in two chronically infected cell lines by preventing the maturation of the multivesicular body, a process that begins in the early endosome and ends with the sorting of cargo to the lysosome. By contrast, knocking down proteins in the retromer complex, which diverts cargo away from the multivesicular body caused an increase in PrPsc levels. These results suggest that the multivesicular body is the major site for intracellular conversion of PrPc to PrPsc.

## INTRODUCTION

Prion diseases or the transmissible spongiform encephalopathies (TSEs) are a group of neurodegenerative disorders of humans and animals characterized by neuronal cell loss, gliosis, spongiosis and deposition of abnormal amyloid protein. The crucial event in TSE pathogenesis is believed to be the conversion of the normal host cellular prion protein, PrPc, to a conformationally altered form, PrPsc, that is closely associated with disease pathogenesis (Prusiner, 1998). The conversion of PrPc to PrPsc causes profound changes in the structure and biochemical properties of PrPsc. PrPc has a high α-helical content, is soluble in detergents and is sensitive to proteolytic digestion by proteinase K, whereas PrPsc is β-sheet rich, insoluble in detergents and shows partial resistance to proteinase K digestion.

PrPc is a GPI-anchored protein, that, after synthesis, is processed in the endoplasmic reticulum (ER) and Golgi before reaching the plasma membrane (Harris, 2003). After PrPc is internalized, it traffics to the early endosome (EE) where it is sorted either to the recycling endosome to be returned to the plasma membrane or to the late endosome/multivesicular body (LE/MVB) to be degraded in the lysosome (Campana et al., 2005). PrPsc appears to traffic along the same endocytic route, although it is degraded at a much slower rate than PrPc (Borchelt et al., 1990). There are still many details of PrPsc cell biology that are not well understood, owing in part to the fact that it is not possible to follow the trafficking of PrPsc in real time. To localize PrPsc in the cell, it has to be denatured prior to immunolabeling (Taraboulos et al., 1995). In addition, conformation-specific antibodies to distinguish PrPsc from PrPc are not available. Therefore, although the basic trafficking pathway has been established, there is still no consensus as to whether PrPsc is internalized through a clathrin-dependent or a clathrin-independent pathway (Goold et al., 2011; Veith et al., 2009). In addition, studies differ both in regard to the steady-state distribution of PrPsc as it traffics along the endocytic pathway and the endosomal compartment where prion conversion occurs. Several studies have proposed that prion conversion occurs as PrPc traffics along the endo-lysosomal pathway (Borchelt et al., 1992; Caughey et al., 1991; Magalhães et al., 2005), whereas some recent studies have proposed that conversion occurs along the endocytic recycling pathway (Goold et al., 2013; Marijanovic et al., 2009). Adding to this lack of consensus, one study has reported that conversion occurs along the trans-Golgi network (TGN) to endoplasmic reticulum retrograde pathway (Béranger et al., 2002). There is also evidence that prion conversion takes place on the plasma membrane (Baron et al., 2006; Goold et al., 2011; Rouvinski et al., 2014).

Owing to the above discrepancies, we have redetermined the major intracellular site of prion conversion using two chronically PrPsc-infected cell lines, SMB and ScN2a. In agreement with earlier studies (Marijanovic et al., 2009; Pimpinelli et al., 2005; Veith et al., 2009), we found that PrPsc localized to endosomes associated with both the endocytic recycling and degradative pathways. Interestingly, in examining the mechanism clearance of PrPsc by calpain inhibitors (Yadavalli et al., 2004), we observed that these inhibitors caused a marked alteration in the localization of PrPsc; PrPsc was predominantly in swollen endosomes that were positive for both LAMP1 and CI-M6PR, characteristic of LE/MVBs, prior to PrPsc clearance. Similarly, inhibiting the maturation of the MVB by knocking down Rab7a, Tsg101 or Hrs (also known as HGS), caused PrPsc to be first localized to aberrant MVBs and then cleared from the cell. In contrast, the cellular level of PrPsc was increased by knocking down proteins in the retromer complex, which inhibits recycling from the MVB. These results suggest that the major internal site of prion conversion is the mature MVB.

## RESULTS

### Cell biology of PrPsc clearance by calpain inhibitors

**Fig. 1.**
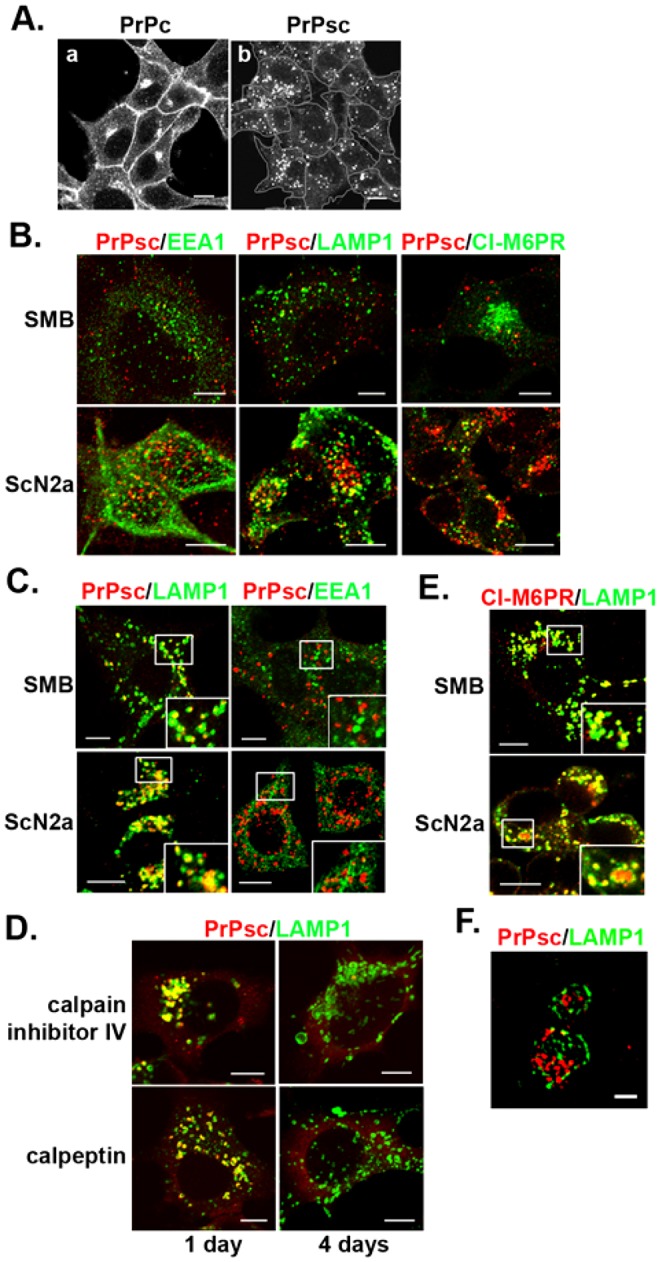
**Effect of calpain inhibitors on the localization of PrPsc in SMB and ScN2a cells.** (A) Immunostaining of SMB cells with anti-PrP antibody before (a) and after (b) 5 M GdnHCl treatment. Cells were immunostained with SAF32 and AH6 antibody to detect both PrPc and PrPsc, respectively. The confocal settings used in imaging PrPsc do not detect PrPc. (B) Immunostaining of SMB and ScN2a cells for PrPsc and different marker proteins in the endocytic pathway in non-treated cells. (C) Effect of overnight incubation of SMB cells with MDL-28170 on PrPsc localization. SMB and ScN2a cells were stained for PrPsc and either LAMP1 or EEA1. (D) Immunostained PrPsc and LAMP1 in SMB cells incubated with either calpain inhibitor IV or calpeptin for the indicated times. (E) Immunostaining of SMB and ScN2a cells for CI-M6PR and LAMP1 following overnight incubation with MDL-28170. Insets are an enlargement of the boxed area. (F) Super-resolution image of PrPsc and LAMP1 in SMB cells incubated overnight with MDL-28170. Scale bars: 10 µm (A–E), 1 µm (F).

One prion clearance event that is not at all understood is the clearing that takes place when PrPsc-infected cells are incubated with calpain inhibitors (Yadavalli et al., 2004). The calpains, a family of Ca^2+^-activated cysteine proteases, are predominantly cytoplasmic proteases (Wang et al., 2005), whereas internalized PrPc and PrPsc traffic on the intraluminal membranes of endosomes. We first examined how calpain inhibitors affect PrPsc localization in two chronically infected cell lines, ScN2a and SMB. Fig. 1A shows that in non-treated cells, PrPc was predominantly on the plasma membrane and at the Golgi, whereas PrPsc had an endosomal localization, which was visualized by treating the cells with guanidine to denature the PrPsc (Taraboulos et al., 1995). Given that there was no detectable staining of the plasma membrane or the Golgi when imaging PrPsc, this indicates that the fluorescence intensity of immunostained PrPsc must be much greater than that of immunostained PrPc, in agreement with previous studies (Marijanovic et al., 2009; Veith et al., 2009). The PrPsc colocalized with markers of the endocytic pathway, such as EEA1, LAMP1, and CI-M6PR (Fig. 1B), as shown previously (Marijanovic et al., 2009; Pimpinelli et al., 2005; Veith et al., 2009). Both SMB and ScN2a cells showed heterogeneity in the number of PrPsc-positive endosomes per cell and in the extent of PrPsc colocalization with a given endosomal marker.

Interestingly, incubating cells with calpain inhibitors, which was previously shown to clear PrPsc (Yadavalli et al., 2004), caused an unusual localization of PrPsc. After overnight incubation with the calpain inhibitor MDL-28170, most of the PrPsc was present in enlarged endosomes, which were positive for LAMP1, a marker of LEs/MVBs and lysosomes, and negative for EEA1, a marker of the EE (Fig. 1C). The extent of colocalization of PrPsc with LAMP1 was initially 20±5% (mean±s.d.), whereas after overnight incubation with MDL-28170, it increased to 83±10%. Two other calpain inhibitors, calpain inhibitor IV and calpeptin, also produced PrPsc-laden swollen LAMP1-positive endosomes prior to PrPsc clearance (Fig. 1D). After 4 days incubation with calpain inhibitors, the LAMP1-positive endosomes remained enlarged, but were now devoid of PrPsc. Therefore, this effect of calpain inhibitors is not due to off-target drug effects. The enlarged LAMP1-positive endosomes persisted even after the PrPsc was cleared, which shows that this effect of calpain inhibitors is independent of PrPsc. Furthermore, the LAMP1-positive endosomes became enlarged when HeLa cells were incubated overnight with MDL-28170, which further shows that the phenotype caused by calpain inhibitors is unrelated to PrPsc (supplementary material Fig. S1). To determine whether the LAMP1-positive endosomes were LE/MVBs or lysosomes, cells were stained with antibody against the cation-independent mannose 6-phosphate receptor (CI-M6PR), which stains LE/MVBs, but not lysosomes (Geuze et al., 1988). Given that the swollen LAMP1-positive endosomes in cells incubated with MDL-28170 were CI-M6PR positive (Fig. 1E), these endosomes are aberrant MVBs and not lysosomes. To obtain higher resolution images of PrPsc in these aberrant MVBs, cells were imaged by super-resolution microscopy. As shown in Fig. 1F, PrPsc was primarily inside the swollen MVBs, suggesting that PrPsc is on intraluminal membranes (supplementary material Movie 1).

**Fig. 2.**
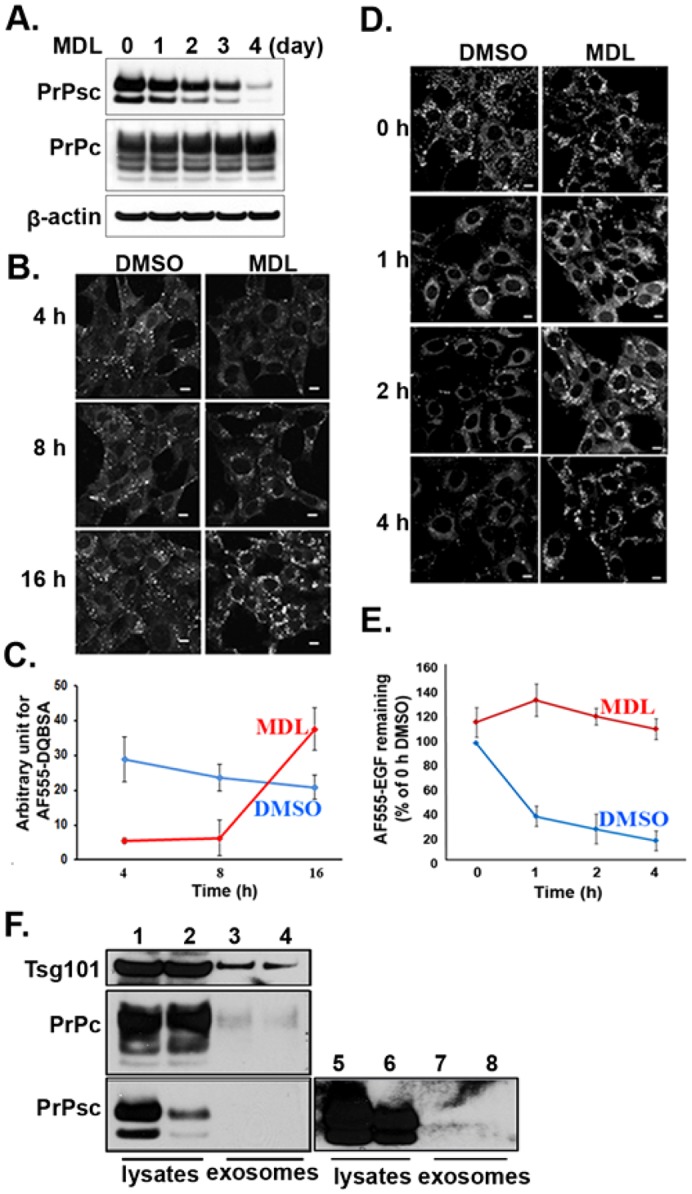
**Clearance of PrPsc by calpain inhibitors is not due to a decrease in cellular PrPc, an increase in endosomal proteolytic activity or the release of PrPsc-laden exosomes.** (A) Western blot of PrPc and PrPsc in cells following different times of incubation with MDL-28170 (50 µM). Actin was used as an internal loading control. (B) DQ-Red–BSA fluorescence monitoring proteolytic activity of the endolysomal compartment. DQ-Red–BSA (10 µg/ml) was internalized for 15 min in SMB cells treated overnight with DMSO (control) or 50 µM MDL. Cells were washed and incubated further in regular medium and imaged at the indicated times. (C) Quantification of the fluorescence intensity of DQ-Red–BSA fluorescence from cells scanned at the indicated times. The DQ-Red fluorescence intensity was measured using Metamorph analysis at the different time points. (D) MDL treatment reduces the rate of degradation of EGF. SMB cells were treated for 1 day either with DMSO (control) or MDL. Both groups of cells were serum starved for 4 h before incubating cells for 15 min with Alexa-Fluor-555–EGF (500 ng/ml). The EGF was then washed out, complete medium was added, and then cells were imaged at the indicated times. (E) Time course of EGF degradation in control and MDL-treated cells. The data were normalized to the intensity of the control cells obtained after washing out the EGF. The EGF intensity was measured using Metamorph analysis at the different time points. Data in C and E are mean±s.d. (*n* = 5). (F) Examination of PrP in exosome-enriched population. A western blot probing for Tsg101, PrPc, and PrPsc in cell lysates and exosome-enriched preparation is shown. After growing SMB cells for 4 days in the absence (lanes 1, 3, 5 and 7) and presence of MDL-28170 (lanes 2, 4, 6 and 8), cells and medium were collected to prepare cell lysates (lanes 1, 2, 5 and 6) and an exosome-enriched population (lanes 3, 4, 7 and 8). The same blot shown in lanes 1–4 were exposed for a longer time (lanes 5–8). Scale bars: 10 µm (B,D).

Because PrPsc was sequestered in enlarged MVBs in cells incubated with MDL-28170, we wanted to examine whether there was an increase in endosomal proteolysis causing a decrease in cellular PrPc, which in turn lead to PrPsc clearance. Western blot analysis showed that incubating SMB cells for 4 days with MDL-28170 did not significantly affect the levels of PrPc (Fig. 2A), whereas it caused a marked reduction in cellular PrPsc, in agreement with a previous study (Yadavalli et al., 2004). It should be noted that to detect the PrPsc on western blots, gels were loaded with ten times more protein than that used to visualize PrPc because PrPsc is only a small percentage of the total PrP present in the cell. Therefore, calpain inhibitors do not clear PrPsc by reducing the level of cellular PrPc.

The above results suggest that the enlarged MVBs present in cells incubated with calpain inhibitors do not have high proteolytic activity. This was confirmed by measuring the endosomal proteolytic activity with DQ-Red BSA fluorescence, a fluorogenic substrate for proteases, which produces a bright fluorescent product when proteolyzed. The control cells showed high fluorescence intensity of DQ-Red BSA after 4 h of internalization, whereas similar incubation of MDL-treated cells failed to show substantial fluorescence intensity, indicating that these endosomes do not have high proteolytic activity (Fig. 2B). In contrast to the control cells, the MDL-treated cells only showed high fluorescence 16 h after internalizing DQ-Red BSA (Fig. 2C). Consistent with this observation, the rate of degradation of Alexa-Fluor-555-conjugated epidermal growth factor (EGF), a lysosomal substrate, was measured in control cells and in cells treated overnight with MDL. As shown in Fig. 2D,E, there was no substantial degradation of EGF in the MDL-treated cells over the 4-h chase time period, whereas most of the EGF was degraded in the control cells. Therefore, clearance of PrPsc is not due to increased proteolytic degradation by the enlarged MVBs.

Another possibility suggested by the presence of the enlarged MVBs is that PrPsc clearance is due to MDL-28170 stimulating the fusion of MVBs with the plasma membrane. This fusion releases the intraluminal vesicles (ILVs) of the MVB as exosomes to the medium. Given that exosomes have previously been shown to contain both PrPc and PrPsc (Fevrier et al., 2004; Leblanc et al., 2006; Veith et al., 2009), stimulated exosome release by MDL-28170 might clear PrPsc. To test this, an exosome-enriched preparation was prepared from the tissue culture medium, which was collected over 4 days from SMB cells grown in the presence and absence of MDL-28170. The levels of PrPc and PrPsc were analyzed in the cell lysates and in the exosome-enriched preparation by western blot analysis (Fig. 2F). In addition, the blots were probed for the protein, Tsg101, a characteristic marker of exosomes (Théry et al., 2009). As expected, the MDL-treated cell lysates had much lower level of PrPsc than the control cells. However, only trace levels of PrPc was present in the exosome-enriched preparation made from the medium of control and MDL-28170-treated cells. Regardless of MDL treatment, negligible amounts of PrPsc were detected in the exosome-enriched preparations even when the western blot was highly overexposed (Fig. 2F, lanes 5–8). Therefore, MDL-28170 treatment does not clear PrPsc by stimulating the release of PrPsc-containing exosomes.

### Effect of knocking down components of the endocytic pathway on PrPsc levels and localization

**Fig. 3.**
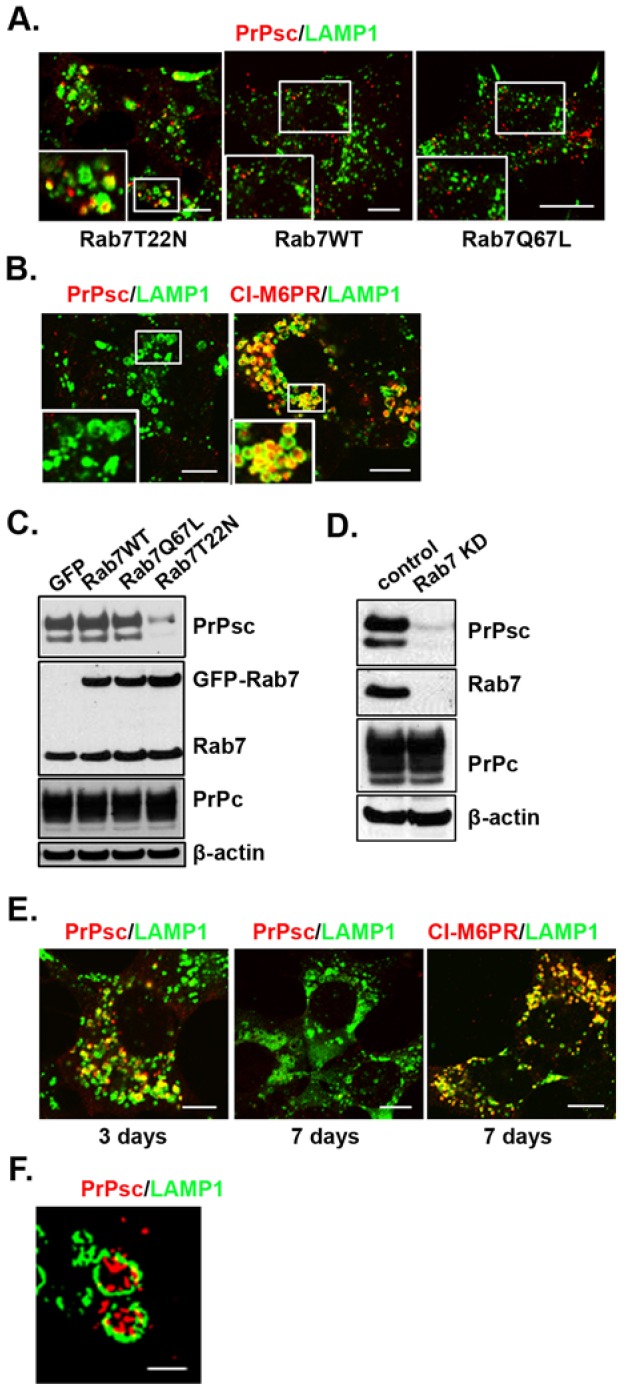
**Clearance of PrPsc is due to lack of maturation of the MVBs.** (A) Effect of overexpressing different Rab7a constructs on the localization of PrPsc and LAMP1. SMB cells were immunostained for PrPsc (red) and LAMP1 (green) at 3 days after transfection with Rab7(T22N), Rab7(WT) or Rab7(Q67L). (B) The stable SMB cell line expressing GFP–Rab7(T22N) clears PrPsc from the MVB. Cells were stained with LAMP1 and either PrPsc or M6PR (red). (C) Western blot of PrPsc and PrPc in lysates from SMB cells stably expressing the indicated GFP–Rab7 constructs. Actin was used as an internal loading control. (D) Western blot of PrPsc and PrPc in lysates from SMB cells depleted of Rab7a. (E) Immunostaining of PrPsc and LAMP1 in SMB cells in cells treated once (day 3) and twice (day 7) with siRNAs oligonucleotides to knockdown Rab7a. (F) Super-resolution image of PrPsc and LAMP1 in SMB cells partially depleted of Rab7. Insets are an enlargement of the boxed area. Scale bars: 10 µm (A,B,E), 1 µm (F).

Because calpain inhibitors redistributed PrPsc to swollen LAMP1-positive endosomes prior to the PrPsc clearance, we examined whether a similar phenotype could be obtained by altering the intracellular trafficking of PrPsc in the cell. To achieve this, we first inhibited Rab7a activity by expressing the dominant-negative Rab7 mutant, Rab7(T22N), given that the loss of Rab7 activity inhibits the maturation of the MVB (Russell et al., 2012). Immunostaining of SMB cells expressing GFP-labeled Rab7(T22N) showed that PrPsc now localized to swollen LAMP1-positive endosomes (Fig. 3A). In contrast, the expression of either GFP-labeled wild-type Rab7 or the constitutively active Rab7 mutant, Rab7(Q67L), did not produce enlarged LE/MVBs or alter the distribution of PrPsc. To determine the long-term effects of expressing these different GFP-labeled Rab7 constructs on PrPsc levels, we made stable cell lines by growing cells under selection conditions for several weeks. The stable Rab7(T22N) cell line showed no detectable PrPsc in the LAMP1- and CI-M6PR-positive endosomes (Fig. 3B). Western blot analysis of the Rab7(T22N) stable cell line, which was more than 80% GFP positive, showed a 75% reduction in PrPsc (Fig. 3C). By contrast, there was no significant change in the PrPsc levels in stable cell lines expressing either wild-type Rab7 or Rab7(Q67L).

Given that overexpression of Rab7 might titrate out other sorting factors, we examined the effect of knocking down Rab7a on PrPsc localization and clearance. Western blots were run on cell lysates that were treated with two rounds of small interfering RNA (siRNA) oligonucleotides (Fig. 3D). Compared to the mock control, the level of PrPsc in the knockdown cells were 8±4% (mean±s.d.; *n* = 4) and 4±3% (*n* = 4) in SMB and ScN2a, respectively. Fig. 3E shows that as the Rab7a is knocked down (3 days after transfecting once with siRNA oligonucleotides), the PrPsc localizes predominantly to enlarged LAMP1- and CI-M6PR positive endosomes. Image analysis showed there was >75% colocalization of the PrPsc with LAMP1-positive endosomes. Imaging of the cells at higher resolution using super-resolution microscopy showed PrPsc inside these aberrant MVBs (Fig. 3F; supplementary material Movie 2). Therefore, the loss of Rab7a activity, which in turn inhibits maturation of the MVB, leads to clearance of PrPsc. These results suggest that maturation of the MVB is necessary for PrPsc propagation.

**Fig. 4.**
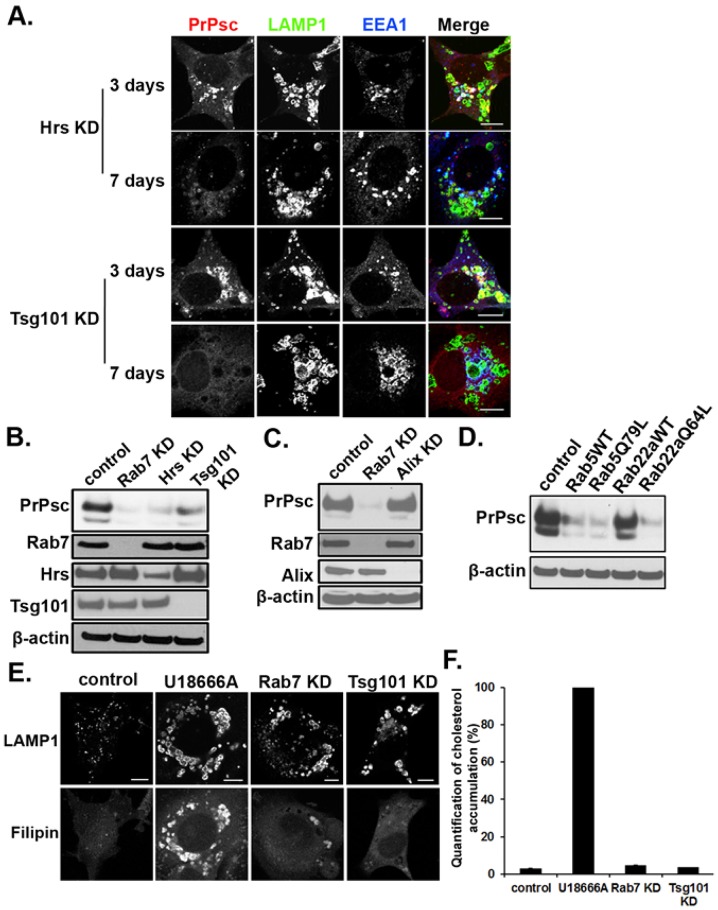
**Knocking down Hrs or Tsg101 alters PrPsc localization and decreases the level of PrPsc.** (A) Hrs or Tsg101 was knocked down (KD) for the indicated times before immunostaining. The merge image shows PrPsc (red), LAMP1 (green) and EEA1 (blue). (B) Western blot of PrPsc and PrPc in lysates from control and in cells knocked down for the following proteins: Rab7a, Hrs, Tsg101. (C) Western blot of PrPsc in lysates from SMB cells depleted of Alix. (D) Western blot of PrPsc in lysates prepared from SMB cells stably expressing the indicated Rab constructs. Immunoblots were probed with antibodies against PrP and actin. (E) Filipin staining of SMB cells grown under different conditions. Control cells, cells incubated in U18666A (50 µM final concentration) for 2 days, Rab7a-knockdown cells, and Tsg101-knockdown cells were stained with filipin and anti-LAMP1 antibody. Scale bar, 10 µm. (F) Relative cholesterol levels under different conditions. Data (mean±s.d., *n* = 10) were normalized by setting the intensity of the background and U18666A-treated cells to 0% and 100%, respectively.

Next, the maturation of the MVB was inhibited by knocking down proteins in the endosomal sorting complexes required for transport (ESCRT) complexes to determine whether this also caused a reduction in PrPsc levels. The maturation of the MVB is dependent on the sequential binding of different ESCRT complexes (Hurley and Emr, 2006; Piper and Katzmann, 2007). To inhibit the maturation process, we knocked down either Hrs, a protein in the ESCRT-0 complex, or Tsg101, a protein in the ESCRT-I complex. Knocking down either one of these ESCRT proteins caused the PrPsc to first localize to swollen LAMP1-positive endosomes, followed by a marked reduction in PrPsc levels (Fig. 4A). The swollen endosomes were also EEA1 positive, indicating the formation of a hybrid organelle. The marked reduction in PrPsc levels caused by knocking down either Hrs or Tsg101 was confirmed by western blot analysis (Fig. 4B); the residual PrPsc level was only 28±9% (*n* = 6, mean±s.d.) in SMB and ScN2a cells compared to the mock treated cells. In contrast to these results and in agreement with a previous study (Marijanovic et al., 2009), PrPsc levels were not significantly reduced by knocking down Alix (Fig. 4C), a multifunctional adaptor protein that interacts with both the ESCRT-I and ESCRT-III complexes (Odorizzi, 2006). In addition, knocking down Alix did not affect the localization of PrPsc (supplementary material Fig. S2A). Our attempts to knockdown Vps4 (both VpsA and Vps4B) were unsuccessful because the cells were not viable. These results show that knocking down core components of the ESCRT machinery, which inhibits maturation of the MVB, caused a marked reduction in PrPsc levels.

If the conversion of PrPc into PrPsc takes place downstream of the early endosome, then inhibiting trafficking from the early endosome should likewise clear PrPsc. The transport of cargo out of the early endosome was inhibited by overexpressing either wild-type Rab5 or the constitutively active Rab5, Rab5(Q71L). Previous studies have shown that expression of either of these constructs, but especially Rab5(Q71L), caused enlargement of the early endosomes (Stenmark et al., 1994) and inhibited both the recycling and degradation of cargo (Huotari and Helenius, 2011). When these constructs were expressed in SMB cells, they produced hybrid organelles that were positive for both EEA1 and LAMP1 staining (supplementary material Fig. S2B). PrPsc localized to these enlarged endosomes, but over time, the level of PrPsc decreased. Swollen hybrid endosomes were also obtained in cells overexpressing wild-type Rab22a or the constitutively active form Rab22, Rab22(Q64L) (Mesa et al., 2001). In the western blot in Fig. 4D, there was ∼75% reduction in PrPsc levels in stable cell lines expressing either wild-type Rab5 or Rab5(Q71L). A similar reduction was observed in the stable cell line overexpressing Rab22a(Q64L), while overexpressing wild-type Rab22a caused ∼50% reduction in PrPsc, as shown previously (Marijanovic et al., 2009). Therefore, inhibiting the trafficking of cargo out of the early endosome causes PrPsc clearance.

Given that perturbations in cellular cholesterol cause clearance of PrPsc (Gilch et al., 2009; Marzo et al., 2013), cells were stained for free cholesterol with filipin. In Fig. 4E, LAMP1 immunostaining and the filipin staining are shown for control, Rab7a-knockdown, Tsg101-knockdown cells and cells treated with U18666A, a drug that blocks the egress of cholesterol from LE/MVBs and lysosomes (Cenedella, 2009). The fluorescence was quantified and then normalized by setting the fluorescence intensity of the U18666A-treated cells to 100% and the background fluorescence to 0% (Fig. 4F). Compared to control cells, the free cholesterol was not significantly affected by knocking down Tsg101, as shown previously (Du et al., 2012). The Rab7a-knockdown cells showed a modest increase (<twofold) in cholesterol, which might be contributing to PrPsc clearance along with lack of maturation of the MVB.

### Effect of knocking down retromer components on PrPsc levels and localization

**Fig. 5.**
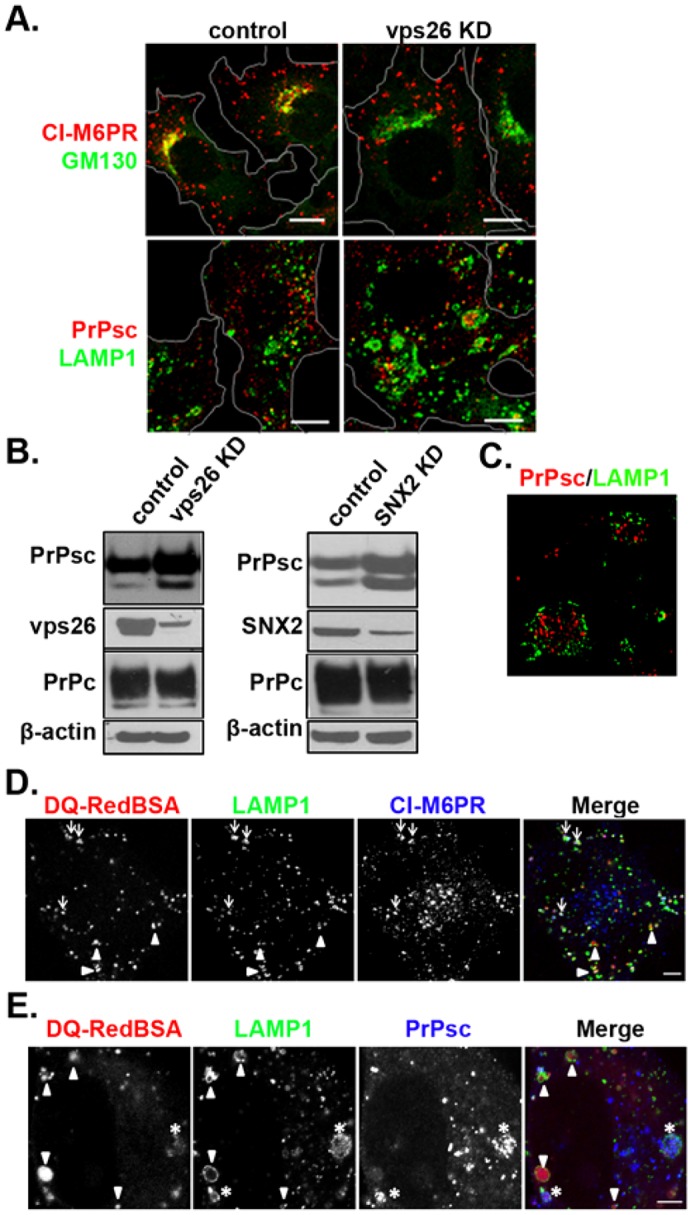
**Knocking down Vps26 alters PrPsc localization and increases the level of PrPsc.** (A) Control and Vps26-knockdown (KD) cells were stained with the indicated antibodies. (B) Western blots of PrPsc and PrPc in lysates from control and Vps26-knockdown cells and SNX-2 knockdown cells. Actin was loaded as an internal loading control. (C) Super-resolution image of PrPsc and LAMP1 in Vps26-knock down cell. (D) Colcalization of the fluorescence endosomal proteolytic indicator DQ-Red–BSA, in control cells with different endosomal markers. Cells were fixed after internalizing DQ-Red–BSA (10 µg/ml) for 4 h at 37°C and then immunostained. The merge image shows proteolyzed DQ-Red–BSA (red), LAMP1 (green) and CI-M6PR (blue). Endosomes with high proteolytic activity that are LAMP1-positive and CI-M6PR-negative, and LAMP-positive and CI-M6PR positive are indicated by the arrowheads and arrows, respectively. (E) PrPsc localizes to MVBs in Vps26-knockdown cells. After internalizing DQ-Red–BSA (red), cells were stained for LAMP1 (green) and PrPsc (blue). The asterisks indicate LAMP1-positive endosomes that are positive for PrPsc and have low DQ-Red–BSA fluorescence. The arrowheads indicate LAMP1-positive endosomes with no detectable PrPsc and high DQ-Red BSA fluorescence. Scale bars: 10 µm (A,D,E), 1 µm (C).

The retromer complex, which is composed of Vps26, Vps29, Vps35 and members of the sorting nexin (SNX) family (Bonifacino and Hurley, 2008) sorts cargo from the MVB to either the plasma membrane or the TGN (Cullen and Korswagen, 2012; Seaman, 2012). Therefore, we examined the effect of knocking down components of the retromer complex to determine the effect of the retromer-dependent trafficking on cellular PrPsc levels. A block in recycling from the MVB would be expected to increase PrPsc levels in the MVB if this is the site of prion conversion. In addition, the degradative capacity of the lysosome is reduced because inhibiting retromer-dependent trafficking leads to improper sorting of lysosomal hydrolyases (Seaman, 2004). Taken together, these two effects lead to the prediction that inhibiting this pathway should cause an increase in the steady-state level of PrPsc. To test this prediction, we knocked down Vps26 and the inhibition of retrograde recycling was confirmed by examining the localization of CI-M6PR with the Golgi (GM130 staining). As shown in Fig. 5A, after knocking down Vps26, CI-M6PR was no longer associated with the Golgi, confirming that this retromer-dependent trafficking of cargo to the TGN was inhibited. In the Vps26-knockdown cells, there were swollen LAMP1-positive endosomes, some of which contained PrPsc. However, unlike the Rab7a-knockdown cells, PrPsc was not restricted to the LAMP1-positive endosomes, indicating that the retromer pathway is only one of the pathways used to recycle PrPsc. As predicted, inhibiting retromer-dependent trafficking caused an increase in PrPsc levels (Fig. 5B). Quantification of the western blots showed that, compared to controls, knocking down Vps26 increased PrPsc to 138±9% (*n* = 3, mean±s.d.) and 125±4 (*n* = 3) in SMB and ScN2a cells, respectively. When we knocked down SNX2 in SMB cells, this increased PrPsc levels to 125±13% (*n* = 3). Cells were not viable when both SNX1 and SNX2 were knocked down. The level of PrPc was not affected by knocking down retromer components, which is consistent with the fact that the total PrPsc in the cell is only a small percentage (>5%) of the total prion protein. Therefore, the increase in PrPsc levels does not reflect in a significant decrease in the PrPc levels.

The swollen LAMP1-positive endosomes containing PrPsc were imaged by super-resolution microscopy. As shown in Fig. 5C, PrPsc was present within these swollen endosomes (supplementary material Movie 3). To determine whether the PrPsc-laden endosomes were MVBs or lysosomes we again used the fluorescence proteolytic DQ-Red BSA because CI-M6PR stained all the LAMP1-positive endosomes in the Vps26-knockdown cells. The DQ-Red BSA fluorescence intensity was standardized by measuring the fluorescence of lysosomes in control cells after internalizing DQ-BSA Red (Fig. 4D). When DQ-Red BSA intensity was measured in Vps26 knockdown cells, PrPsc was present in LAMP1-positive endosomes with low proteolytic activity, indicating these structures are MVBs (Fig. 4E). Taken together, these data suggest that in the Vps26-knockdown cells, PrPsc localizes on the intraluminal vesicles (ILVs) of the MVBs.

## DISCUSSION

Inhibiting maturation of the MVB by using different cell biological tools caused a marked reduction in PrPsc levels both in SMB and ScN2a cells, but did not significantly affect PrPc levels. These results are not compatible with the identification of the recycling endosome as the major internal conversion site for prion conversion (Marijanovic et al., 2009). Interestingly, the data in that study are quite similar to ours even though we reached very different conclusion. In agreement with Marijanovic et al. (Marijanovic et al., 2009), we did not observe a reduction in PrPsc when Alix was knocked down, which led them to rule out the MVB as a site for prion conversion. However, Alix is not an essential component of the MVB maturation pathway; it is only required for the maturation of a subset of MVBs (Odorizzi, 2006) and its depletion does not affect the degradation of the EGF receptor, a lysosomal cargo (Cabezas et al., 2005).

Our observation that calpain inhibitors caused clearance of PrPsc by forming aberrant MVBs led us to examine cell biological tools that produce a similar phenomenon. However, the role of calpains in the maturation of the MVB is not understood, but it is unrelated to the presence of PrPsc. In general, calpains are cytosolic proteases, but they do bind to membranes with high affinity (Hood et al., 2004). In addition, calpain has been found in the lumen of the ER and Golgi (Hood et al., 2004; Hood et al., 2006) and, more recently, in the lumen of the MVB (Rintanen et al., 2012). It is therefore possible that inhibiting calpain activity not only prevents endoproteolytic cleavage of PrPsc, but also cleavage of other cargos, which would explain why calpain inhibition causes swelling of the LE/MVB. One proteolytic substrate of calpains is the tyrosine phosphatase HD-PTP (PTPN23), which is a member of the Bro1 domain family (Castiglioni and Maier, 2012). HD-PTP has been shown to have an important role in maturation of the MVB and in the vectoral movement of cargo through the ESCRT pathway (Ali et al., 2013; Doyotte et al., 2008), which might explain the inhibition of the maturation of the MVB by calpain inhibitors.

How do we account for the marked reduction in PrPsc levels when maturation of the MVB is inhibited? Both PrPc and PrPsc are known to traffic along the endo-lysosomal pathway with these proteins finally being degraded in the lysosome. Because the cellular level of PrPsc is determined by both its rate of degradation and its rate of conversion of PrPc, inhibiting MVB maturation must either increase the rate of PrPsc degradation or reduce its rate of conversion from PrPc. Our current results show that MDL-treatment reduces degradation of lysosomal cargo. Likewise, it has previously been shown that inhibiting the maturation of the MVB reduces the degradation of lysosomal cargo (Raiborg et al., 2008; Razi and Futter, 2006). Therefore, increased degradation of PrPsc cannot explain the clearance of PrPsc when MVB maturation is inhibited. Instead, the decrease in cellular PrPsc must be due to a reduction in the rate of conversion of PrPc into PrPsc. With a reduction in the rate of conversion, clearance would then occur upon dilution of PrPsc upon cell division.

**Fig. 6.**
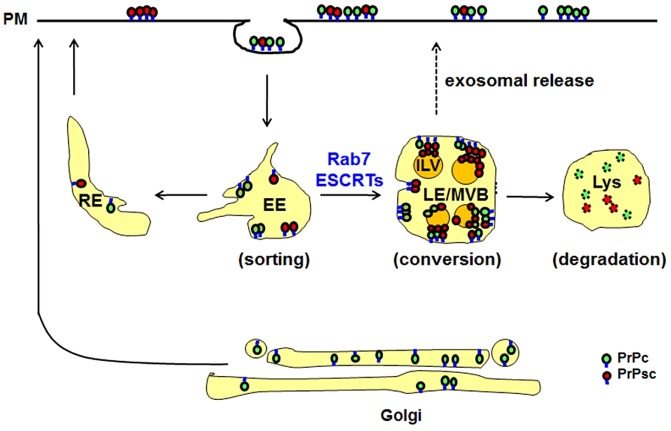
**Model of cellular trafficking of PrPc and PrPsc.** Following the internalization of PrP and PrPsc, these proteins are sorted in the early endosome (EE) either to be recycled to the plasma membrane via the recycling endosome (RE) or to traffic along the endolysosomal pathway. PrPc is converted into PrPsc in the MVB and the PrPsc is either recycled back to the plasma membrane or degraded in the lysosome (Lys). Recycling to the plasma membrane (dashed arrows) occurs via a retromer-dependent pathway, but it is not yet clear whether cargo is transferred to the TGN prior to reaching the plasma membrane. PrPsc is also released into the medium when the MVB fuses with the plasma membrane to release its intraluminal vesicles as exosomes.

Our results support the model shown in Fig. 6, which shows that the mature MVB is the major internal site of PrPsc conversion. This site of conversion is consistent both with the reduction in PrPsc when MVB maturation is inhibited and the increase in PrPsc when retromer-dependent trafficking is inhibited. Although retromer has a role in recycling, it is not clear which retromer-dependent sorting pathway is being used to recycle PrPsc. In addition to the retromer complex recycling cargo to the TGN (Bonifacino and Hurley, 2008), it also recycles cargo to the plasma membrane (Steinberg et al., 2013; Temkin et al., 2011). In fact, the retromer complex has been shown to export cargo directly from the late endosome to the plasma membrane (Hesketh et al., 2014). Importantly, PrPc and PrPsc can traffic from the early endosome back to the plasma membrane through alternative pathways, but as for the major site of internal conversion of PrPc to PrPsc, our data show that this occurs in the MVB and not the recycling endosome, as reported previously (Marijanovic et al., 2009). Of course, this does not mean that conversion does not occur on the plasma membrane, which could be a primary site of conversion when cells are infected with scrapie from external sources (Goold et al., 2011; Rouvinski et al., 2014).

Previous studies have shown by immunogold labeling that PrPc and PrPsc are present on the ILVs (Fevrier et al., 2004; Veith et al., 2009). Typically, ubiquitylated cargo is sorted into the ILVs, but given that the ILVs are very cholesterol rich (Möbius et al., 2003), PrPc and PrPsc might sort with cholesterol-rich lipid rafts. In the MVB, the outer membrane surface of the ILV faces the inner membrane surface of the MVB, which is the same geometry occurs when PrPsc infects neighboring cells by cell contact and when cells are infected by exosomes or microsomes from scrapie-infected brains. This geometry might favor conversion by the trans-interaction of PrPsc with PrPc inducing the unfolding of PrPc into a misfolded intermediate conformation prior to the formation of the PrPsc. Alternatively, conversion might occur by cis-interaction between PrPc and PrPsc with the role of the MVB to concentrate the PrPc and PrPsc due to the high cholesterol content of the ILVs. In a recent study, a large proportion of the total prion protein on the plasma membrane was shown to be nascent PrPsc that formed when the cells were infected with PrPsc (Rouvinski et al., 2014). The geometry for the conversion that occurs on the plasma membrane upon external infection might be similar to that occurring within the MVB.

The MVB apparently has multiple roles in prion disease. First, it is the major site of intracellular conversion of PrPc to PrPsc. Second, the MVB has recently been shown to be the site of *de novo* generation of PrPsc when N2a cells are infected with purified PrPsc fibers (Yamasaki et al., 2014). Finally, it is important for PrPsc propagation based on the finding that when MVBs fuse with the plasma membrane, they release exosomes containing PrPc and PrPsc (Fevrier et al., 2004; Veith et al., 2009). Exosomes from PrPsc-infected cells have been shown to infect cultured neuronal cells with PrPsc (Alais et al., 2008; Leblanc et al., 2006), but not SMB cells (Kanu et al., 2002). Therefore, our finding that the mature MVB is the major site of conversion has important consequences with regard to the pathogenesis of mad cow disease and perhaps other neurodegenerative diseases that have been shown to occur through prion-like transmission. In the future, the ESCRTs and Rab7, as well as Vsp26, might be of interest as relevant drug targets for the treatment of neurodegenerative diseases.

## MATERIALS AND METHODS

### Antibodies

The following mouse antibodies were used: anti-Rab7 (Sigma), anti-Tsg101 (GeneTex), anti-β-actin (Abcam), anti-GM130 (BD Transduction Laboratories) and anti-prion (SAF32, Cayman chemical; AH6, TSE Resource Center,). The following rabbit antibodies were used: anti-Hrs (Novus Biologicals), anti-TGN38 (AbD Serotec), anti-GFP (Abcam), anti-EEA1 (Cell Signaling), anti-Vps26 (a gift from Juan Bonifacino, Cell Biology Metabolism Program, NICHD, NIH, Bethesda, MD), anti-CI-M6PR (a gift from Linton Traub, Department of Cell Biology, University of Pittsburgh, PA) and anti-Alix (Bethyl Laboratories). Rat anti-LAMP1 antibody (Developmental Studies Hybridoma Bank) was used. PrPc and PrPsc were routinely detected using DyL488, Cy3 and DyL647-conjugated secondary antibodies (Jackson ImmunoResearch Laboratories). Western blots were probed using horseradish peroxidase (HRP)-conjugated secondary antibodies (Jackson ImmunoResearch Laboratories) and InfraRed Dye 680 and 800 secondary antibodies (Li-Cor Bioscience).

### Chemicals and plasmids

The calpain inhibitors (50 µM final concentration) were: MDL-28170 (Enzo Life Sci.), calpeptin (Enzo Life Sci.) and calpain inhibitor IV (EMD Millipore). U18666A was from Biomol Research Laboratories and siRNA oligomers were either from Dharmacon Thermo Scientific or Santa Cruz Biotechnology. Alexa-Fluor-555-conjugated EGF and DQ-Red BSA were from Life Technologies.

### Cell lines

Scrapie-infected mouse brain (SMB) were maintained in DMEM/high glucose/GlutaMAX (catalog number 10569; Life Technologies) with 10% FBS, 100 U/ml penicillin and 100 µg/ml streptomycin. Scrapie-infected N2a (ScN2a-22L) cells were cultured in OPTI-MEM (Life Technologies) with 10% FBS, 100 U/ml penicillin and 100 µg/ml streptomycin. Stable cells lines of SMB expressing different GFP–Rab constructs were made by growing cells in G418 antibiotic (Life Technologies) for several months. The cells were maintained in antibiotic to maintain selection. The stable cell lines had greater than 80% GFP-positive cells.

### Transfection

Plasmids were transfected using X-tremeGENE HP DNA transfection reagent (Roche Applied Science). The medium was replaced the next day with fresh medium containing the selection marker G418. Cells were maintained in the presence of G418 for a minimum of 6 weeks to make the stable cell lines. For knockdown experiments using siRNA oligonucleotides, the cells were reversely transfected with 20 nM siRNA oligomers twice at 3-day intervals using Lipofectamine RNAiMAX reagent (Life Technologies). On the day 7, the cells were either harvested for western blotting or fixed for immunostaining.

### Immunofluorescence and western blotting

Cells plated onto Lab-Tek glass chamber slides (Nalge Nunc) or round glass coverslips (Electron Microscopy Sciences) were fixed in 4% PFA for 10 min and washed three times with PBS containing 10% FBS. Prior to immunostaining PrPsc within the cell, the fixed cells were treated with 5 M GdnHCl for 5 min to denature the proteins (Taraboulos et al., 1995). For immunostaining and immunoblotting, SAF32 and AH6 antibodies were used to detect PrPc and PrPsc, respectively. When cells were co-stained for PrPsc and other endosomal marker proteins, the endosomal marker protein was stained with primary and secondary antibodies, followed by fixation with 4% PFA. PrPsc and then denatured with 5 M GdnHCl prior to immunostaining. For western blots, 50 µg whole-cell lysate was loaded to each well except for PrPsc. To detect PrPsc by western blotting, 500 µg of cell lysates was digested with 5 µl of Proteinase K (2 mg/ml, Life Technologies) in a final volume 500 µl at 37°C for 1 h. After stopping the reaction with PMSF (Sigma), the insoluble Proteinase-K-resistant proteins were collected by ultracentrifugation at 100,000 ***g*** for 1 h in a TL100 centrifuge (Beckman). The pellet was resuspended in PBS for SDS-PAGE. Protein concentrations were determined by using the BCA Protein Assay Reagent (Pierce). Western blots were performed according to standard procedures. PrPsc was detected by using ECL chemiluminescence (Thermo Scientific). The other proteins on the western blots were detected using the Odyssey infrared system (Li-Cor Bioscience). Quantification of the western blots was performed using the Odyssey analysis program.

### Filipin staining

To detect the free cholesterol in the cells, cells were fixed with 3% PFA for 1 h and stained with 0.05 mg/ml filipin (Sigma) dissolved in PBS containing 10% FBS. Filipin staining was imaged using a UV filter set (340–380 nm excitation, 40 nm dichroic, 430 nm long pass filter).

### DQ-Red-BSA and EGF degradation assay

To evaluate the protease activity in MDL-treated cells, we used DQ-Red BSA, which is a fluorogenic substrate for protease so it produces bright fluorescent products upon hydrolysis, and Alexa-Fluor-555–EGF. After cells were treated with DMSO or MDL for a day, the cells were serum starved for 4 h prior to loading of DQ-Red–BSA or EGF. Then, for the DQ-Red–BSA degradation assay, 10 µg/ml DQ-Red–BSA was loaded for 15 min and chased for the indicated time. For the EGF degradation assay, 500 ng/ml Alexa-Fluor-555–EGF was loaded for 15 min at 37°C and chased for the indicated times.

### Imaging and data analysis

Confocal images were obtained with the Zeiss LSM 510 or the Zeiss LSM 780 microscope using a 63×, 1.4 NA objective. The identical confocal settings were used when comparing the relative fluorescence intensity between images. Super-resolution images were obtained with the Delta Vision Omx microscope (GE Health Care) using a 60× 1.42 NA objective. The super-resolution data were processed using ImageJ software. The MetaMorph colocalization application (Molecular Devices) was used to quantify colocalization between proteins in individual cells.

### Preparation of exosome-enriched fraction

First, cell culture supernatant was collected and cell debris and dead cells were removed by centrifugation at 10,000 ***g*** for 30 min. The resulting supernatant was then ultracentrifuged at 100,000 ***g*** for 1 h to collect the small vesicles. The pellets was washed in a large volume of PBS to eliminate contaminating proteins and centrifuged again at 100,000 ***g*** for 1 h to pellet the exosome-enriched fraction (Thery et al., 2006).

## Supplementary Material

Supplementary Material

## References

[b1] Alais S. Simoes S. Baas D. Lehmann S. Raposo G. Darlix J. L. Leblanc P. (2008). Mouse neuroblastoma cells release prion infectivity associated with exosomal vesicles. Biol. Cell 100, 603–618 10.1042/BC20080025 18422484

[b2] Ali N. Zhang L. Taylor S. Mironov A. Urbé S. Woodman P. (2013). Recruitment of UBPY and ESCRT exchange drive HD-PTP-dependent sorting of EGFR to the MVB. Curr. Biol. 23, 453–461. 23477725 10.1016/j.cub.2013.02.033

[b3] Baron G. S. Magalhães A. C. Prado M. A. Caughey B. (2006). Mouse-adapted scrapie infection of SN56 cells: greater efficiency with microsome-associated versus purified PrP-res. J. Virol. 80, 2106–2117 10.1128/JVI.80.5.2106-2117.2006 16474119 PMC1395383

[b4] Béranger F. Mangé A. Goud B. Lehmann S. (2002). Stimulation of PrP(C) retrograde transport toward the endoplasmic reticulum increases accumulation of PrP(Sc) in prion-infected cells. J. Biol. Chem. 277, 38972–38977 10.1074/jbc.M205110200 12163492

[b5] Bonifacino J. S. Hurley J. H. (2008). Retromer. Curr. Opin. Cell Biol. 20, 427–436 10.1016/j.ceb.2008.03.009 18472259 PMC2833274

[b6] Borchelt D. R. Scott M. Taraboulos A. Stahl N. Prusiner S. B. (1990). Scrapie and cellular prion proteins differ in their kinetics of synthesis and topology in cultured cells. J. Cell Biol. 110, 743–752 10.1083/jcb.110.3.743 1968466 PMC2116048

[b7] Borchelt D. R. Taraboulos A. Prusiner S. B. (1992). Evidence for synthesis of scrapie prion proteins in the endocytic pathway. J. Biol. Chem. 267, 16188–16199. 1353761

[b8] Cabezas A. Bache K. G. Brech A. Stenmark H. (2005). Alix regulates cortical actin and the spatial distribution of endosomes. J. Cell Sci. 118, 2625–2635 10.1242/jcs.02382 15914539

[b9] Campana V. Sarnataro D. Zurzolo C. (2005). The highways and byways of prion protein trafficking. Trends Cell Biol. 15, 102–111 10.1016/j.tcb.2004.12.002 15695097

[b10] Castiglioni S. Maier J. A. (2012). The tyrosine phosphatase HD-PTP (PTPN23) is degraded by calpains in a calcium-dependent manner. Biochem. Biophys. Res. Commun. 421, 380–383 10.1016/j.bbrc.2012.04.024 22510412

[b11] Caughey B. Raymond G. J. Ernst D. Race R. E. (1991). N-terminal truncation of the scrapie-associated form of PrP by lysosomal protease(s): implications regarding the site of conversion of PrP to the protease-resistant state. J. Virol. 65, 6597–6603. 1682507 10.1128/jvi.65.12.6597-6603.1991 PMC250721

[b12] Cenedella R. J. (2009). Cholesterol synthesis inhibitor U18666A and the role of sterol metabolism and trafficking in numerous pathophysiological processes. Lipids 44, 477–487 10.1007/s11745-009-3305-7 19440746

[b13] Cullen P. J. Korswagen H. C. (2012). Sorting nexins provide diversity for retromer-dependent trafficking events. Nat. Cell Biol. 14, 29–37 10.1038/ncb2374 22193161 PMC3613977

[b14] Doyotte A. Mironov A. McKenzie E. Woodman P. (2008). The Bro1-related protein HD-PTP/PTPN23 is required for endosomal cargo sorting and multivesicular body morphogenesis. Proc. Natl. Acad. Sci. USA 105, 6308–6313 10.1073/pnas.0707601105 18434552 PMC2359801

[b15] Du X. Kazim A. S. Brown A. J. Yang H. (2012). An essential role of Hrs/Vps27 in endosomal cholesterol trafficking. Cell Reports 1, 29–35 10.1016/j.celrep.2011.10.004 22832105

[b16] Fevrier B. Vilette D. Archer F. Loew D. Faigle W. Vidal M. Laude H. Raposo G. (2004). Cells release prions in association with exosomes. Proc. Natl. Acad. Sci. USA 101, 9683–9688 10.1073/pnas.0308413101 15210972 PMC470735

[b17] Geuze H. J. Stoorvogel W. Strous G. J. Slot J. W. Bleekemolen J. E. Mellman I. (1988). Sorting of mannose 6-phosphate receptors and lysosomal membrane proteins in endocytic vesicles. J. Cell Biol. 107, 2491–2501 10.1083/jcb.107.6.2491 2849607 PMC2115678

[b18] Gilch S. Bach C. Lutzny G. Vorberg I. Schätzl H. M. (2009). Inhibition of cholesterol recycling impairs cellular PrP(Sc) propagation. Cell. Mol. Life Sci. 66, 3979–3991 10.1007/s00018-009-0158-4 19823766 PMC2777232

[b19] Goold R. Rabbanian S. Sutton L. Andre R. Arora P. Moonga J. Clarke A. R. Schiavo G. Jat P. Collinge J. (2011). Rapid cell-surface prion protein conversion revealed using a novel cell system. Nat. Commun. 2, 281 10.1038/ncomms1282 21505437 PMC3104518

[b20] Goold R. McKinnon C. Rabbanian S. Collinge J. Schiavo G. Tabrizi S. J. (2013). Alternative fates of newly formed PrPSc upon prion conversion on the plasma membrane. J. Cell Sci. 126, 3552–3562 10.1242/jcs.120477 23813960 PMC3744024

[b21] Harris D. A. (2003). Trafficking, turnover and membrane topology of PrP. Br. Med. Bull. 66, 71–85 10.1093/bmb/66.1.71 14522850

[b22] Hesketh G. G. Pérez-Dorado I. Jackson L. P. Wartosch L. Schäfer I. B. Gray S. R. McCoy A. J. Zeldin O. B. Garman E. F. Harbour M. E. (2014). VARP is recruited on to endosomes by direct interaction with retromer, where together they function in export to the cell surface. Dev. Cell 29, 591–606 10.1016/j.devcel.2014.04.010 24856514 PMC4059916

[b23] Hood J. L. Brooks W. H. Roszman T. L. (2004). Differential compartmentalization of the calpain/calpastatin network with the endoplasmic reticulum and Golgi apparatus. J. Biol. Chem. 279, 43126–43135 10.1074/jbc.M408100200 15302874

[b24] Hood J. L. Brooks W. H. Roszman T. L. (2006). Subcellular mobility of the calpain/calpastatin network: an organelle transient. BioEssays 28, 850–859 10.1002/bies.20440 16927317

[b25] Huotari J. Helenius A. (2011). Endosome maturation. EMBO J. 30, 3481–3500 10.1038/emboj.2011.286 21878991 PMC3181477

[b26] Hurley J. H. Emr S. D. (2006). The ESCRT complexes: structure and mechanism of a membrane-trafficking network. Annu. Rev. Biophys. Biomol. Struct. 35, 277–298 10.1146/annurev.biophys.35.040405.102126 16689637 PMC1648078

[b27] Kanu N. Imokawa Y. Drechsel D. N. Williamson R. A. Birkett C. R. Bostock C. J. Brockes J. P. (2002). Transfer of scrapie prion infectivity by cell contact in culture. Curr. Biol. 12, 523–530 10.1016/S0960-9822(02)00722-4 11937020

[b28] Leblanc P. Alais S. Porto-Carreiro I. Lehmann S. Grassi J. Raposo G. Darlix J. L. (2006). Retrovirus infection strongly enhances scrapie infectivity release in cell culture. EMBO J. 25, 2674–2685 10.1038/sj.emboj.7601162 16724107 PMC1500854

[b29] Magalhães A. C. Baron G. S. Lee K. S. Steele-Mortimer O. Dorward D. Prado M. A. Caughey B. (2005). Uptake and neuritic transport of scrapie prion protein coincident with infection of neuronal cells. J. Neurosci. 25, 5207–5216 10.1523/JNEUROSCI.0653-05.2005 15917460 PMC6724812

[b30] Marijanovic Z. Caputo A. Campana V. Zurzolo C. (2009). Identification of an intracellular site of prion conversion. PLoS Pathog. 5, e1000426 10.1371/journal.ppat.1000426 19424437 PMC2673690

[b31] Marzo L. Marijanovic Z. Browman D. Chamoun Z. Caputo A. Zurzolo C. (2013). 4-hydroxytamoxifen leads to PrPSc clearance by conveying both PrPC and PrPSc to lysosomes independently of autophagy. J. Cell Sci. 126, 1345–1354 10.1242/jcs.114801 23418355

[b32] Mesa R. Salomón C. Roggero M. Stahl P. D. Mayorga L. S. (2001). Rab22a affects the morphology and function of the endocytic pathway. J. Cell Sci. 114, 4041–4049. 11739636 10.1242/jcs.114.22.4041

[b33] Möbius W. van Donselaar E. Ohno-Iwashita Y. Shimada Y. Heijnen H. F. Slot J. W. Geuze H. J. (2003). Recycling compartments and the internal vesicles of multivesicular bodies harbor most of the cholesterol found in the endocytic pathway. Traffic 4, 222–231 10.1034/j.1600-0854.2003.00072.x 12694561

[b34] Odorizzi G. (2006). The multiple personalities of Alix. J. Cell Sci. 119, 3025–3032 10.1242/jcs.03072 16868030

[b35] Pimpinelli F. Lehmann S. Maridonneau-Parini I. (2005). The scrapie prion protein is present in flotillin-1-positive vesicles in central- but not peripheral-derived neuronal cell lines. Eur. J. Neurosci. 21, 2063–2072 10.1111/j.1460-9568.2005.04049.x 15869502

[b36] Piper R. C. Katzmann D. J. (2007). Biogenesis and function of multivesicular bodies. Annu. Rev. Cell Dev. Biol. 23, 519–547 10.1146/annurev.cellbio.23.090506.123319 17506697 PMC2911632

[b37] Prusiner S. B. (1998). Prions. Proc. Natl. Acad. Sci. USA 95, 13363–13383 10.1073/pnas.95.23.13363 9811807 PMC33918

[b38] Raiborg C. Malerød L. Pedersen N. M. Stenmark H. (2008). Differential functions of Hrs and ESCRT proteins in endocytic membrane trafficking. Exp. Cell Res. 314, 801–813 10.1016/j.yexcr.2007.10.014 18031739

[b39] Razi M. Futter C. E. (2006). Distinct roles for Tsg101 and Hrs in multivesicular body formation and inward vesiculation. Mol. Biol. Cell 17, 3469–3483 10.1091/mbc.E05-11-1054 16707569 PMC1525239

[b40] Rintanen N. Karjalainen M. Alanko J. Paavolainen L. Mäki A. Nissinen L. Lehkonen M. Kallio K. Cheng R. H. Upla P. (2012). Calpains promote α2β1 integrin turnover in nonrecycling integrin pathway. Mol. Biol. Cell 23, 448–463 10.1091/mbc.E11-06-0548 22160595 PMC3268724

[b41] Rouvinski A. Karniely S. Kounin M. Moussa S. Goldberg M. D. Warburg G. Lyakhovetsky R. Papy-Garcia D. Kutzsche J. Korth C. (2014). Live imaging of prions reveals nascent PrPSc in cell-surface, raft-associated amyloid strings and webs. J. Cell Biol. 204, 423–441 10.1083/jcb.201308028 24493590 PMC3912534

[b42] Russell M. R. Shideler T. Nickerson D. P. West M. Odorizzi G. (2012). Class E compartments form in response to ESCRT dysfunction in yeast due to hyperactivity of the Vps21 Rab GTPase. J. Cell Sci. 125, 5208–5220 10.1242/jcs.111310 22899724 PMC3533395

[b43] Seaman M. N. (2004). Cargo-selective endosomal sorting for retrieval to the Golgi requires retromer. J. Cell Biol. 165, 111–122 10.1083/jcb.200312034 15078902 PMC2172078

[b44] Seaman M. N. (2012). The retromer complex – endosomal protein recycling and beyond. J. Cell Sci. 125, 4693–4702 10.1242/jcs.103440 23148298 PMC3517092

[b45] Steinberg F. Gallon M. Winfield M. Thomas E. C. Bell A. J. Heesom K. J. Tavaré J. M. Cullen P. J. (2013). A global analysis of SNX27-retromer assembly and cargo specificity reveals a function in glucose and metal ion transport. Nat. Cell Biol. 15, 461–471 10.1038/ncb2721 23563491 PMC4052425

[b46] Stenmark H. Parton R. G. Steele-Mortimer O. Lütcke A. Gruenberg J. Zerial M. (1994). Inhibition of rab5 GTPase activity stimulates membrane fusion in endocytosis. EMBO J. 13, 1287–1296. 8137813 10.1002/j.1460-2075.1994.tb06381.x PMC394944

[b47] Taraboulos A. Scott M. Semenov A. Avrahami D. Laszlo L. Prusiner S. B. (1995). Cholesterol depletion and modification of COOH-terminal targeting sequence of the prion protein inhibit formation of the scrapie isoform. J. Cell Biol. 129, 121–132 10.1083/jcb.129.1.121 7698979 PMC2120366

[b48] Temkin P. Lauffer B. Jäger S. Cimermancic P. Krogan N. J. von Zastrow M. (2011). SNX27 mediates retromer tubule entry and endosome-to-plasma membrane trafficking of signalling receptors. Nat. Cell Biol. 13, 715–721 10.1038/ncb2252 21602791 PMC3113693

[b49] Thery C. Amigorena S. Raposo G. Clayton A. (2006). Isolation and characterization of exosomes from cell culture supernatants and biological fluids. Curr. Protoc. Cell Biol. Chapter 3, Unit 3 22 10.1002/0471143030.cb0322s30 18228490

[b50] Théry C. Ostrowski M. Segura E. (2009). Membrane vesicles as conveyors of immune responses. Nat. Rev. Immunol. 9, 581–593 10.1038/nri2567 19498381

[b51] Veith N. M. Plattner H. Stuermer C. A. Schulz-Schaeffer W. J. Bürkle A. (2009). Immunolocalisation of PrPSc in scrapie-infected N2a mouse neuroblastoma cells by light and electron microscopy. Eur. J. Cell Biol. 88, 45–63 10.1016/j.ejcb.2008.08.001 18834644

[b52] Wang X. Wang F. Sy M. S. Ma J. (2005). Calpain and other cytosolic proteases can contribute to the degradation of retro-translocated prion protein in the cytosol. J. Biol. Chem. 280, 317–325 10.1074/jbc.M410649200 15525638

[b53] Yadavalli R. Guttmann R. P. Seward T. Centers, A. P., Williamson, R. A. and Telling, G. C(2004). Calpain-dependent endoproteolytic cleavage of PrPSc modulates scrapie prion propagation. J. Biol. Chem. 279, 21948–21956 10.1074/jbc.M400793200 15026410

[b54] Yamasaki T. Baron G. S. Suzuki A. Hasebe R. Horiuchi M. (2014). Characterization of intracellular dynamics of inoculated PrP-res and newly generated PrP(Sc) during early stage prion infection in Neuro2a cells. Virology 450-451, 324–335 10.1016/j.virol.2013.11.007 24503096 PMC4167762

