# Supplementary material for: The multivesicular body is the major internal site of prion conversion
Source: J Cell Sci. 2015 Apr 1;128(7):1434–43. doi: 10.1242/jcs.165472 (PMC4379730; doi:10.1242/jcs.165472)
Supplement: Supplementary Material [file supp_128_7_1434__index.html]

The multivesicular body is the major internal site of prion conversion — Supplementary Material 

# The multivesicular body is the major internal site of prion conversion

## JCS165472 Supplementary Material

**Files in this Data Supplement:**

- **Supplementary Material**
