# Supplementary material for: The multivesicular body is the major internal site of prion conversion
Source: J Cell Sci. 2015 Apr 1;128(7):1434–43. doi: 10.1242/jcs.165472 (PMC4379730; doi:10.1242/jcs.165472)
Supplement: Supplementary Material [file supp_128.7.1434_JCS165472.pdf]

Supplemental Material:

Figure S1. Treatment of Hela cells with the calpain inhibitor, MDL-28170. HeLa cells were stained for LAMP1 in control (DMSO) and after overnight incubation with MDL-28170. Mouse monoclonal antibody against LAMP1 (H4A3) was from Developmental Studies Hybridoma Bank (Univ. Iowa). The dashed line shows the outline of the cells.

Figure S2. Effect of knocking down Alix and overexpressing Rabs on PrPsc and endosomal structure. A, PrPsc localization and PrPsc levels after knocking down Alix. Control and Alix-knockdown SMB cells were immunostained for PrPsc (red) and LAMP1 (green). B. Effect of expressing different Rab constructs on endosomal structure. SMB cells expressing the indicated Rab construct (green) were immunostained with antibodies against EEA1 (red) and LAMP1 (blue). Scale bar= 10 μm in all panels.

Movie 1: Super-resolution movie of z-stack projection showing PrPsc with the swollen Lamp-1 positive endosomes after treating SMB cells overnight with MDL-28170. Cells were immunostained for Lamp1 (green) and PrPsc (red).

Movie 2. Super-resolution movie of z-stack projection showing PrPsc with the swollen Lamp-1 positive endosomes after knocking down Rab7. Three days after treating SMB cells one time with siRNA oligonucleotides to knockdown Rab7, cells were immunostained for Lamp1 (green) and PrPsc (red).

Movie 3. Super-resolution movie of z-stack projection showing PrPsc with the swollen Lamp-1 positive endosomes after knocking down Vps26. After treating SMB cells two timew with siRNA oligonucleotides to knockdown Vps26, cells were immunostained for Lamp1 (green) and PrPsc (red).

Sup.1

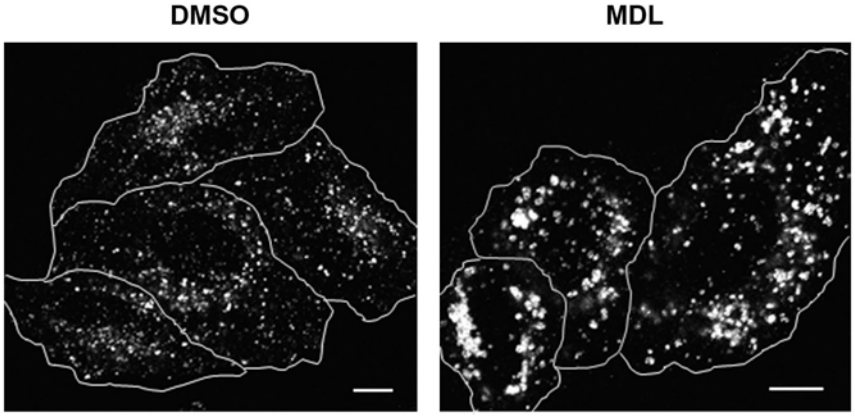

Sup.2

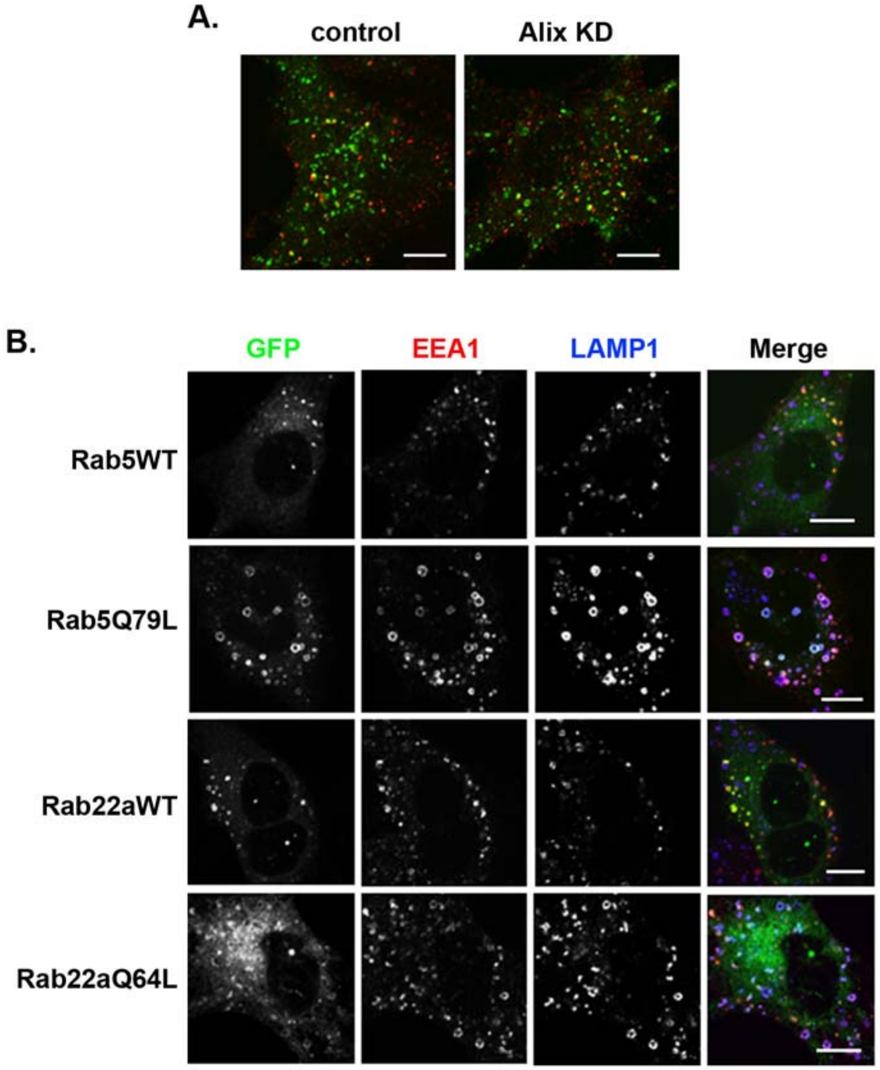
A.
control
Alix KD
B.
GFP
EEA1
LAMP1
Merge
Rab5WT
Rab5Q79L
Rab22aWT
Rab22aQ64L

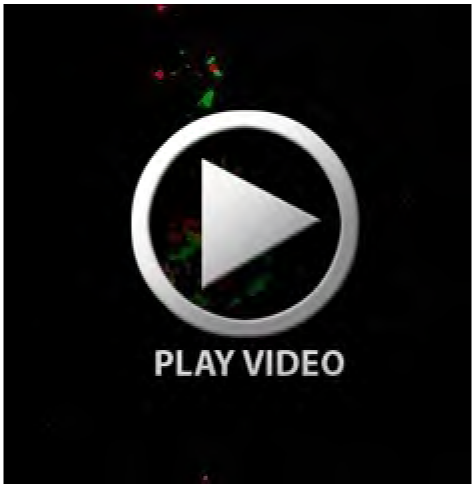

Movie 1.

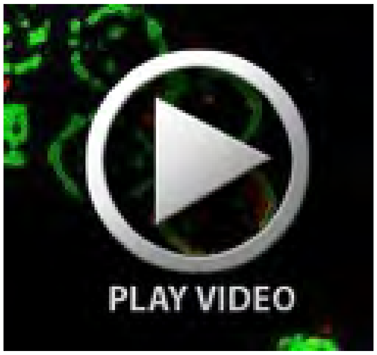

Movie 2.

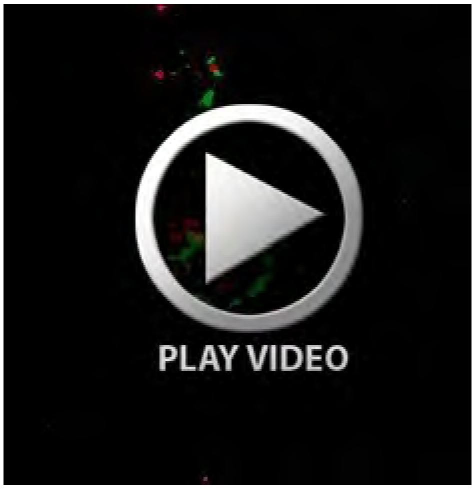

Movie 3.
